# Chromothripsis and DNA Repair Disorders

**DOI:** 10.3390/jcm9030613

**Published:** 2020-02-25

**Authors:** Lusine Nazaryan-Petersen, Victoria Alexandra Bjerregaard, Finn Cilius Nielsen, Niels Tommerup, Zeynep Tümer

**Affiliations:** 1 Department of Cellular and Molecular Medicine, University of Copenhagen, 2200 Copenhagen, Denmark; lusine.nazaryan-petersen@regionh.dk (L.N.-P.); ntommerup@sund.ku.dk (N.T.); 2 Center for Genomic Medicine, Rigshospitalet, 2100 Copenhagen, Denmark; finn.cilius.nielsen@regionh.dk; 3 Kennedy Center, Department of Clinical Genetics, Copenhagen University Hospital, Rigshospitalet, 2600 Glostrup, Denmark; victoria.bjerregaard@regionh.dk; 4 Department of Clinical Medicine, University of Copenhagen, 2200 Copenhagen, Denmark

**Keywords:** chromothripsis, structural variants, DNA repair, DNA repair disorders, DNA double-strand breaks (DSBs), ataxia telangiectasia mutated (*ATM*), ataxia telangiectasia and Rad3-related (*ATR*), *TP53*, micronuclei, chromosome pulverization

## Abstract

Chromothripsis is a mutational mechanism leading to complex and relatively clustered chromosomal rearrangements, resulting in diverse phenotypic outcomes depending on the involved genomic landscapes. It may occur both in the germ and the somatic cells, resulting in congenital and developmental disorders and cancer, respectively. Asymptomatic individuals may be carriers of chromotriptic rearrangements and experience recurrent reproductive failures when two or more chromosomes are involved. Several mechanisms are postulated to underlie chromothripsis. The most attractive hypothesis involves chromosome pulverization in micronuclei, followed by the incorrect reassembly of fragments through DNA repair to explain the clustered nature of the observed complex rearrangements. Moreover, exogenous or endogenous DNA damage induction and dicentric bridge formation may be involved. Chromosome instability is commonly observed in the cells of patients with DNA repair disorders, such as ataxia telangiectasia, Nijmegen breakage syndrome, and Bloom syndrome. In addition, germline variations of *TP53* have been associated with chromothripsis in sonic hedgehog medulloblastoma and acute myeloid leukemia. In the present review, we focus on the underlying mechanisms of chromothripsis and the involvement of defective DNA repair genes, resulting in chromosome instability and chromothripsis-like rearrangements.

## 1. Chromothripsis

**Figure 1:**
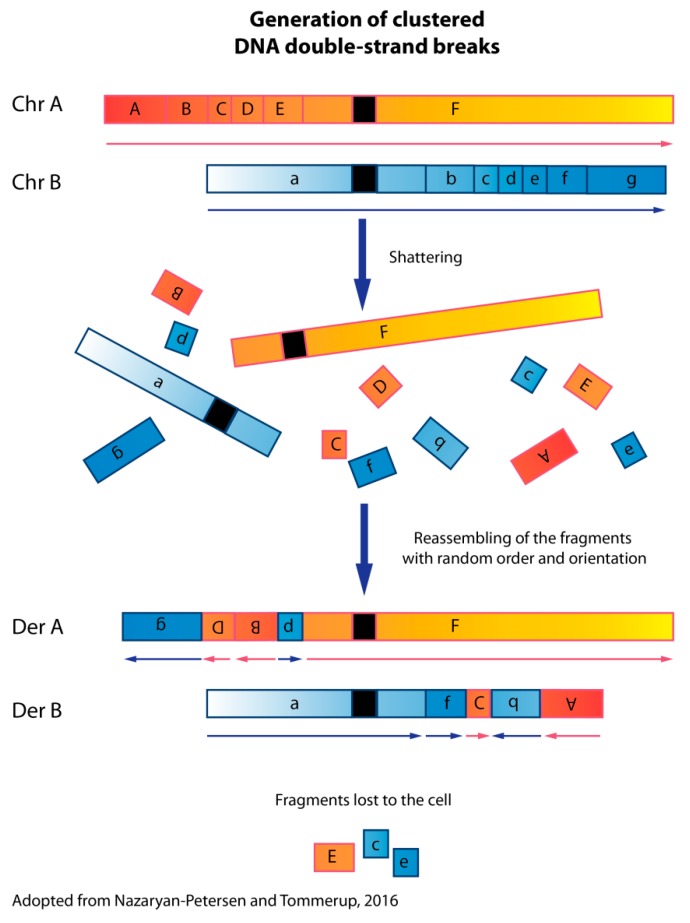
Schematic mechanism of chromothripsis. The first step of chromothripsis is the generation of clustered DNA double-strand breaks. Chromothripsis may involve one or a few chromosomes, a chromosomal arm (both p and q arms), or an entire chromosome. This results in multiple fragments that are stitched together in a random order and orientation by DNA repair machineries. During this process, some of the fragments may be lost. The derivative chromosome(s) will contain complex structural rearrangements. By piecing together all the structural variants detected by paired-end or mate-pair sequencing, it should be possible to delineate the derivative chromosomes.

The mutational mechanism, termed chromothripsis, leading to complex genomic structural rearrangements in confined genomic regions, owes its identification to the development of genome-wide sequencing technologies [1]. Chromothripsis is characterized by local “shattering” or the generation of clustered DNA double-strand breaks (DSBs) involving one or multiple chromosomes and random reassembly of the generated fragments (Figure 1). During the reassembly process, fragments may be deleted, while duplications are almost completely absent. Thus, chromothripsis is distinguished by (1) clustered breakpoints; (2) the oscillation of copy number states between one (deleted fragments with loss of heterozygosity) and two (with maintained heterozygosity); (3) rearrangements affecting a single haplotype (one of two homologous chromosomes); (4) the random order and orientation of the DNA fragments within the derivative chromosomes; and (5) the ability to “walk” through the derivative chromosome by joining the breakpoints if all the breakpoints are available [2].

**Figure 2:**
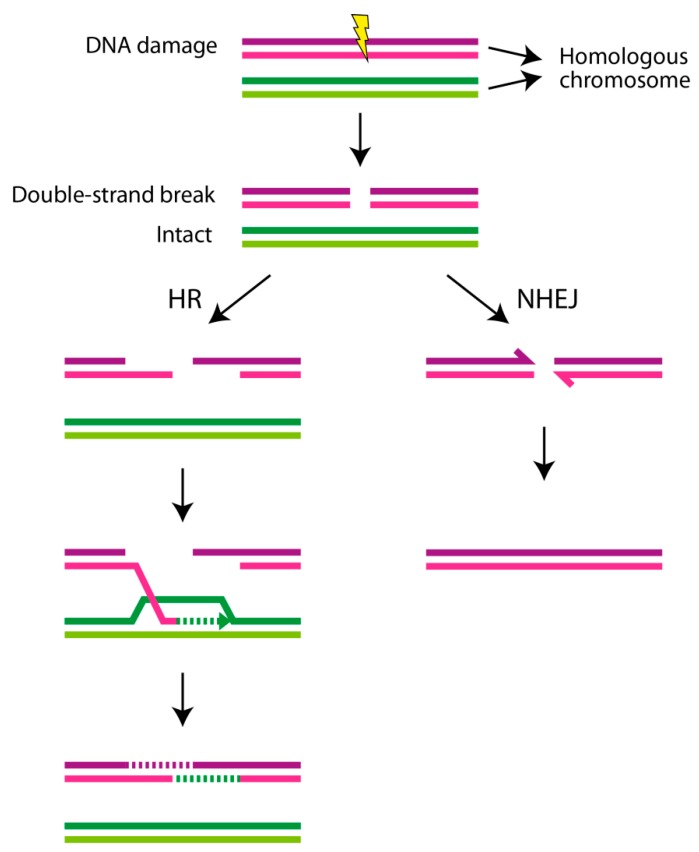
Double-strand breaks (DSBs) and repair mechanisms. Genotoxic factors, such as ionizing radiation, reactive oxygen species, and toxic environmental chemicals lead to DNA damage, which is different from a mutation occurring during DNA replication. Of the different types of DNA lesions, double-strand breakage is the most deleterious form of DNA damage. DSBs are repaired through different DNA repair pathways. Two main forms of DSB repair are homologous recombination (HR), which is an error-free DNA repair, and nonhomologous end joining (NHEJ), which is an error-prone DNA repair. When DSBs are unrepaired, e.g., in HR-mediated DNA repair disorders as described in the text, this leads to cellular transformation, senescence, and/or cell death.

The detailed analysis of the breakpoint junction sequences shows few base-pair microhomologies, if any, short deletions/duplications, and short templated or nontemplated insertions, indicating that for most of the cases, nonhomologous end joining (NHEJ) [3] and/or microhomology-mediated end joining (MMEJ) [4] are the most likely DNA repair mechanisms underlying the gluing process of the generated fragments (Figure 2). However, in rare cases, homologous repeats such as SINE (short interspersed nuclear elements) or LINE (long interspersed nuclear elements) elements may also mediate chromothripsis, as they contain potential L1 endonuclease cleavage sites, resulting in DSBs, and could also subsequently mediate DNA repair via homologous recombination (HR) within the regions [5]. 

## 2. Chromothripsis and Micronucleus Model

**Figure 3:**
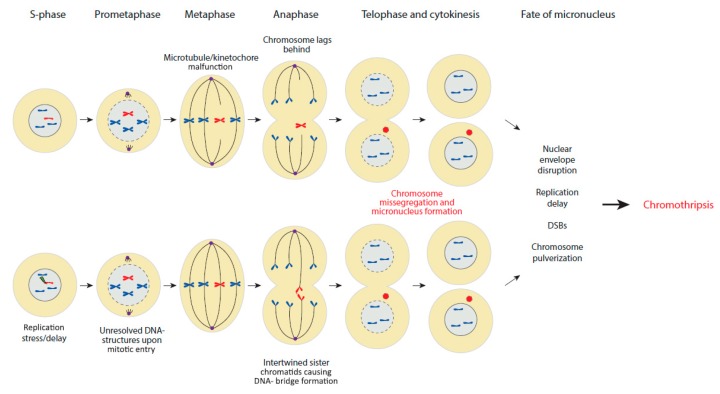
Micronucleus formation during mitotic cell division. A micronucleus can form when a chromosome lags in anaphase, resulting in missegregation and exclusion from the main nucleus upon cytokinesis. This can, for example, occur when the bipolar mitotic spindle fails to capture and segregate chromosomes because of microtubule/kinetochore malfunction (above) or when sister chromatids are entangled throughout mitosis by unresolved replication intermediates (arising in S-phase) that persist as DNA bridges (below) and prohibit faithful segregation. Chromosomes entrapped in micronuclei are accompanied by an unstable nuclear envelope and show delayed replication and susceptibility to DSBs and pulverization. These dramatic mitotic segregation errors are proposed to lead to the dramatic chromosomal rearrangements observed in chromothripsis.

Several hallmarks observed in chromothriptic chromosomes suggest that these complex rearrangements occur within a single or few subsequent cell cycle(s) rather than occurring progressively over multiple cell divisions [1]. The cause of the localized DSBs within relatively small regions is yet unclear, and several hypotheses including ionizing radiation [1,6], the breakage–fusion–bridge cycle associated with telomere attrition [1,7], aborted apoptosis [8], as well as endogenous endonucleases [5] have been proposed to play a role. The most favored hypothesis is the formation of a micronucleus, an extranuclear structure with a lipid envelope, following missegregation of a chromosome (micronucleus model) [9,10] (Figure 3). Chromosome segregation errors during mitotic cell division are known causes of aneuploidy, and they are probably also involved in the formation of structural chromosome variations [11]. Missegregation may occur when microtubules fail to capture chromosomes or when the sister chromatids remain entangled at the mitotic entry, leading to the formation of DNA bridges which prevent chromosome(s) from proper segregation [12] (Figure 3). The lagging chromosomes are hereby isolated from the main nucleus and encapsulated in a micronucleus, where they may undergo pulverization as well as asynchronous replication [9] and premature condensation [13].

The association between micronuclei and DNA damage, and genomic instability, is well documented, but the exact sources of DNA damage in micronuclei remain largely unknown. Replication stress may have a dual role of initiating micronuclei formation and promoting DSBs within the micronucleus once it has been formed. The delayed/stalled replication observed in micronuclei may cause a large number of unresolved replication intermediates, which are known to trigger endonuclease-dependent DSB formation [14]; and upon mitotic entry (following disruption of the micronuclear/nuclear membranes), the DNA damage response pathway will then promote the repair of the massive DSBs, leading to chromothripsis.

Another explanation for the massive DSBs in the chromosome(s) within the micronuclei could be the disruption of the micronuclear envelope. This may expose the encapsulated DNA to potential harmful components of the cytoplasm, followed by chromosome fragmentation through DSB formation [10,15]. Upon the breakdown of the envelope, the shattered chromosome(s) of the disrupted micronuclei are reincorporated into the main nucleus and the fragments are rejoined [16]. In chromothripsis, almost all the chromosome fragments are rejoined and only few regions, if any, are lost. One notable observation is that rearrangements are restricted to the missegregated chromosome(s).

## 3. Chromothripsis and Disease

Since chromothripsis involves widespread genomic regions, it may affect a number of completely different diseases [17]. Chromothripsis was first described in a patient with chronic lymphocytic leukemia in 2011 [1], and it has subsequently been observed in a number of tumor types [18,19,20,21,22,23]. Germline chromothripsis with relatively milder complexity level has also been reported in patients with different congenital or developmental disorders [24,25,26,27,28,29,30,31]. Notably, chromothripsis may also be found in asymptomatic individuals and is associated with the truncation or deletion of many protein-coding genes [32,33]. However, such carriers have a high risk of spontaneous abortions or infertility. In addition, there is a report of one somatic chromothripsis event where the deletion of a gain of function mutated autosomal dominant *CXCR4* chemokine receptor gene served as a rescue mechanism and healed the patient with an autoimmune disorder (warts, hypogammaglobulinemia, infections, and myelokathexis (WHIM) syndrome) [34].

## 4. DNA Repair Mechanisms and DNA Damage Response

DNA repair mechanisms play an essential role in maintaining genome integrity and stability. Different DNA repair pathways have evolved to defend mammalian cells from various types of DNA damage, such as pyrimidine dimers, A–G or T–C mismatches, and single-strand or double-strand breaks caused by endogenous and exogenous factors. These DNA lesions are recognized and corrected by specific DNA repair mechanisms, e.g., mismatch repair (MMR) corrects the base–base mismatches and insertion/deletion mispairings generated during DNA replication and recombination [35]; base-excision repair is responsible for repairing single-strand breaks (SSBs) [36]; and HR and NHEJ/MMEJ repair DSBs [37] (Figure 2). DSBs are considered to be the most hazardous type of DNA damage, as incorrect repair may result in chromosomal translocations or other structural rearrangements underlying tumorigenesis. Cells respond to DSBs through complex repair and signaling mechanisms, termed DNA damage response (DDR) [38]. The three key components recognizing DSBs during the early stages of DDR are DNA-dependent protein kinase (DNA-PK), ataxia telangiectasia mutated (ATM), and ataxia telangiectasia and Rad3-related (ATR) protein kinases [38].

## 5. DNA Repair Disorders and Chromothripsis

DNA repair disorders are a heterogeneous group of monogenic diseases where one of the DNA repair pathways (e.g., MMR, HR, or NHEJ) is disrupted. Depending on the impaired repair system, the individual may become prone to cancer susceptibility, neurological disorders, or premature aging due to an accumulation of DNA damage. Chromothripsis may both be a consequence and cause of DNA repair disorders. For example, chromothripsis is a frequent feature in various cancer types associated with germline variants of tumor suppressor or DNA repair genes. Germline variants in *TP53* are strongly associated with chromothripsis in sonic hedgehog medulloblastoma and acute myeloid leukemia, suggesting that *TP53* variants may predispose to chromothripsis [20]. Chromothripsis has also been suggested as a common mechanism leading to genomic rearrangements in colorectal cancer, leading to the deletion or truncation of several tumor suppressor or other cancer-related genes [23]; however, it was not specified whether the patients had germline variants in the MMR genes (*MLH1*, *MSH2*, *MSH6*, and *PMS2*), which are commonly associated with hereditary nonpolyposis colorectal cancer [39]. To our knowledge, defects in MMR genes are not causes but consequences of chromothripsis which lead to the deletion/truncation of these genes, resulting in colorectal cancer. Moreover, genes involved in homologous recombination (HR)-mediated DNA repair, such as *BRCA1*, *BRCA2*, and *RAD51D*, have been associated with an increased risk of breast/ovarian [40] or prostate cancer [41]; and in one yet unpublished study (preprint is available via bioRxiv) that investigated 2658 human cancers, chromothripsis was found to underlie 1.9% of the losses of DNA repair genes, including *MLH1*, *BRCA1*, and *BRCA2* [42].

Another DNA repair disorder with genomic instability, micronuclei formation, and chromothripsis is ataxia telangiectasia (AT, OMIM #208900), which is characterized by cerebellar ataxia, telangiectasia, immune deficiency, and a predisposition to cancer. Like most DNA repair disorders, AT is an autosomal recessive disorder caused by compound heterozygous or homozygous variants in the *ATM* gene that encodes a kinase involved in HR-mediated DNA repair. Ratnaparkhe et al. identified a high frequency of micronuclei and chromothripsis in AT-associated tumor cells and suggested that these features were related to the underlying pathogenic *ATM* variants [43].

AT belongs to the group of DNA repair disorders where the defective genes encode for proteins involved in the HR-mediated DNA repair pathway [44], and there are other monogenic disorders belonging to this group with increased micronuclei formation. A phenomenon similar to chromothripsis has been observed in the autosomal recessive Seckel syndrome 1 (SCKL1, OMIM #210600), caused by homozygous and compound heterozygous mutation in *ATR*. SCKL1 is characterized by severe intrauterine growth deficiency, dwarfism, microcephaly, lymphoma (in few cases), but not immunodeficiency or ataxia [45]. Alderton et al. observed that the cells from patients with SCKL1 showed increased formation of micronuclei as a response to UV or other reagents, causing replication stalling [46]. Notably, the authors observed a phenomenon they named “nuclear fragmentation” that resembled the previously described phenotype of mitotic catastrophe [47,48,49]. This publication is from 2004, long before the designation of the term chromothripsis in 2011 [1]. It is thus plausible that SCKL1 cells may also exhibit chromothripsis, and this should be investigated further with genome-wide sequencing technologies. In addition, we have reported a three-generation family including 11 carriers of germline chromothripsis, where *ATR* was one of the truncated genes, though the other allele was intact [32]. Notably, all the offspring of a grandparent that we could trace the chromothripsis back to were carriers, and none had a normal karyotype. Based on this observation, we hypothesized that a reduced activity of ATR in the germ cells due to monoallelic truncation via chromothripsis may lead to proliferative advantage against the normal cells [32].

Bloom syndrome (BLM, OMIM **#**210900) is another DNA repair disorder; it is characterized by pre- and post-natal growth retardation, microcephaly, hypo- and hyper-pigmented telangiectatic skin which is sensitive to sunlight, and a predisposition to cancer. BLM cells show genomic instability with an increased exchange between sister chromatids and homologous chromosomes. BLM is an autosomal recessive disorder caused by compound heterozygous or homozygous variants in RecQ protein-like 3 (*RECQL3*). *RECQL3* encodes an enzyme (helicase) which functions in the last step of the HR-mediated DNA repair pathway [50]. Besides increased sister chromatid exchange, the BLM cells show increased sensitivity to DSB-causing agents [51], formation of ultrafine bridges [52], and elevated levels of micronuclei which are further exacerbated in the presence of replication stress [53]. Under normal conditions, RECQL3 binds to the ultrafine bridges and facilitates their resolution [52]. In its absence, sister chromatid entanglements persist, leading to the missegregation of chromosomes followed by micronuclei formation. To our knowledge, chromothripsis has not been reported in BLM patients, even though increased genomic rearrangements have been reported in both human BLM cells and in animal BLM models, including *Drosophila* and mouse [50,54,55,56].

The last monogenic disorder in this group is the autosomal recessive Nijmegen breakage syndrome (NBS, OMIM #251260), which is characterized by microcephaly, growth retardation, immunodeficiency, and a predisposition to cancer. The defective gene, *NBN*, encodes for a protein that is part of a DSB repair complex of the same HR pathway. A study from 2000 showed increased micronuclei formation, but chromothripsis was not reported [57].

In conclusion, chromothripsis can be not only a consequence but also the cause of damage within DNA repair pathways. As defective DDR and micronuclei formation are frequently associated with chromothripsis, it is possible that chromothripsis occurs in some of the affected cells in disorders with a defective HR-mediated DNA repair pathway as described for AT and possibly for SCKL1. Further studies of patient cells with genome-wide sequencing technologies, such as whole genome sequencing and mate-pair sequencing, are necessary to answer this question.

## References

[1] Stephens P.J., Greenman C.D., Fu B., Yang F., Bignell G.R., Mudie L.J., Pleasance E.D., Lau K.W., Beare D., Stebbings L.A. (2011). Massive genomic rearrangement acquired in a single catastrophic event during cancer development. Cell.

[2] Korbel J.O., Campbell P.J. (2013). Criteria for inference of chromothripsis in cancer genomes. Cell.

[3] Lieber M.R. (2010). The mechanism of double-strand DNA break repair by the nonhomologous DNA end-joining pathway. Annu. Rev. Biochem..

[4] McVey M., Lee S.E. (2008). MMEJ repair of double-strand breaks (director’s cut): Deleted sequences and alternative endings. Trends Genet..

[5] Nazaryan-Petersen L., Bertelsen B., Bak M., Jonson L., Tommerup N., Hancks D.C., Tumer Z. (2016). Germline Chromothripsis Driven by L1-Mediated Retrotransposition and Alu/Alu Homologous Recombination. Hum. Mutat..

[6] Morishita M., Muramatsu T., Suto Y., Hirai M., Konishi T., Hayashi S., Shigemizu D., Tsunoda T., Moriyama K., Inazawa J. (2016). Chromothripsis-like chromosomal rearrangements induced by ionizing radiation using proton microbeam irradiation system. Oncotarget.

[7] Maciejowski J., Li Y., Bosco N., Campbell P.J., De Lange T. (2015). Chromothripsis and Kataegis Induced by Telomere Crisis. Cell.

[8] Tubio J.M.C., Estivill X. (2011). Cancer: When catastrophe strikes a cell. Nature.

[9] Crasta K., Ganem N.J., Dagher R., Lantermann A.B., Ivanova E.V., Pan Y., Nezi L., Protopopov A., Chowdhury D., Pellman D. (2012). DNA breaks and chromosome pulverization from errors in mitosis. Nature.

[10] Zhang C.-Z., Spektor A., Cornils H., Francis J.M., Jackson E.K., Liu S., Meyerson M., Pellman D. (2015). Chromothripsis from DNA damage in micronuclei. Nature.

[11] Janssen A., van der Burg M., Szuhai K., Kops G.J.P.L., Medema R.H. (2011). Chromosome segregation errors as a cause of DNA damage and structural chromosome aberrations. Science.

[12] Fenech M., Kirsch-Volders M., Natarajan A.T., Surralles J., Crott J.W., Parry J., Norppa H., Eastmond D.A., Tucker J.D., Thomas P. (2011). Molecular mechanisms of micronucleus, nucleoplasmic bridge and nuclear bud formation in mammalian and human cells. Mutagenesis.

[13] Johnson R.T., Rao P.N. (1970). Mammalian cell fusion: Induction of premature chromosome condensation in interphase nuclei. Nature.

[14] Falquet B., Rass U. (2019). Structure-specific endonucleases and the resolution of chromosome underreplication. Genes.

[15] Liu S., Kwon M., Mannino M., Yang N., Renda F., Khodjakov A., Pellman D. (2018). Nuclear envelope assembly defects link mitotic errors to chromothripsis. Nature.

[16] Sudmant P.H., Rausch T., Gardner E.J., Handsaker R.E., Abyzov A., Huddleston J., Zhang Y., Ye K., Jun G., Fritz M.H.-Y. (2015). An integrated map of structural variation in 2,504 human genomes. Nature.

[17] Nazaryan-Petersen L., Tommerup N. (2016). Chromothripsis and Human Genetic Disease. eLS.

[18] Notta F., Chan-Seng-Yue M., Lemire M., Li Y., Wilson G.W., Connor A.A., Denroche R.E., Liang S.-B., Brown A.M.K., Kim J.C. (2016). A renewed model of pancreatic cancer evolution based on genomic rearrangement patterns. Nature.

[19] Molenaar J.J., Koster J., Zwijnenburg D.A., van Sluis P., Valentijn L.J., van der Ploeg I., Hamdi M., van Nes J., Westerman B.A., van Arkel J. (2012). Sequencing of neuroblastoma identifies chromothripsis and defects in neuritogenesis genes. Nature.

[20] Rausch T., Jones D.T.W., Zapatka M., Stutz A.M., Zichner T., Weischenfeldt J., Jager N., Remke M., Shih D., Northcott P.A. (2012). Genome sequencing of pediatric medulloblastoma links catastrophic DNA rearrangements with TP53 mutations. Cell.

[21] Scarpa A., Chang D.K., Nones K., Corbo V., Patch A.-M., Bailey P., Lawlor R.T., Johns A.L., Miller D.K., Mafficini A. (2017). Whole-genome landscape of pancreatic neuroendocrine tumours. Nature.

[22] Fraser M., Sabelnykova V.Y., Yamaguchi T.N., Heisler L.E., Livingstone J., Huang V., Shiah Y.-J., Yousif F., Lin X., Masella A.P. (2017). Genomic hallmarks of localized, non-indolent prostate cancer. Nature.

[23] Kloosterman W.P., Hoogstraat M., Paling O., Tavakoli-Yaraki M., Renkens I., Vermaat J.S., van Roosmalen M.J., van Lieshout S., Nijman I.J., Roessingh W. (2011). Chromothripsis is a common mechanism driving genomic rearrangements in primary and metastatic colorectal cancer. Genome Biol..

[24] Kloosterman W.P., Guryev V., van Roosmalen M., Duran K.J., de Bruijn E., Bakker S.C.M., Letteboer T., van Nesselrooij B., Hochstenbach R., Poot M. (2011). Chromothripsis as a mechanism driving complex de novo structural rearrangements in the germline. Hum. Mol. Genet..

[25] Kloosterman W.P., Tavakoli-Yaraki M., Van Roosmalen M.J., Van Binsbergen E., Renkens I., Duran K., Ballarati L., Vergult S., Giardino D., Hansson K. (2012). Constitutional Chromothripsis Rearrangements Involve Clustered Double-Stranded DNA Breaks and Nonhomologous Repair Mechanisms. Cell Rep..

[26] Chiang C., Jacobsen J.C., Ernst C., Hanscom C., Heilbut A., Blumenthal I., Mills R.E., Kirby A., Lindgren A.M., Rudiger S.R. (2012). Complex reorganization and predominant non-homologous repair following chromosomal breakage in karyotypically balanced germline rearrangements and transgenic integration. Nat. Genet..

[27] Genesio R., Fontana P., Mormile A., Casertano A., Falco M., Conti A., Franzese A., Mozzillo E., Nitsch L., Melis D. (2015). Constitutional chromothripsis involving the critical region of 9q21.13 microdeletion syndrome. Mol. Cytogenet..

[28] Nazaryan L., Stefanou E.G., Hansen C., Kosyakova N., Bak M., Sharkey F.H., Mantziou T., Papanastasiou A.D., Velissariou V., Liehr T. (2014). The strength of combined cytogenetic and mate-pair sequencing techniques illustrated by a germline chromothripsis rearrangement involving FOXP2. Eur. J. Hum. Genet..

[29] Slamova Z., Nazaryan-Petersen L., Mehrjouy M.M., Drabova J., Hancarova M., Marikova T., Novotna D., Vlckova M., Vlckova Z., Bak M. (2018). Very short DNA segments can be detected and handled by the repair machinery during germline chromothriptic chromosome reassembly. Hum. Mutat..

[30] Nazaryan-Petersen L., Oliveira I.R., Mehrjouy M.M., Mendez J.M.M., Bak M., Bugge M., Kalscheuer V.M., Bache I., Hancks D.C., Tommerup N. (2019). Multigenic truncation of the semaphorin-plexin pathway by a germline chromothriptic rearrangement associated with Moebius syndrome. Hum. Mutat..

[31] Eisfeldt J., Pettersson M., Vezzi F., Wincent J., Kaller M., Gruselius J., Nilsson D., Syk Lundberg E., Carvalho C.M.B., Lindstrand A. (2019). Comprehensive structural variation genome map of individuals carrying complex chromosomal rearrangements. PLoS Genet..

[32] Bertelsen B., Nazaryan-Petersen L., Sun W., Mehrjouy M.M., Xie G., Chen W., Hjermind L.E., Taschner P.E.M., Tümer Z. (2015). A germline chromothripsis event stably segregating in 11 individuals through three generations. Genet. Med..

[33] De Pagter M.S., Van Roosmalen M.J., Baas A.F., Renkens I., Duran K.J., Van Binsbergen E., Tavakoli-Yaraki M., Hochstenbach R., Van Der Veken L.T., Cuppen E. (2015). Chromothripsis in healthy individuals affects multiple protein-coding genes and can result in severe congenital abnormalities in offspring. Am. J. Hum. Genet..

[34] McDermott D.H., Gao J.L., Liu Q., Siwicki M., Martens C., Jacobs P., Velez D., Yim E., Bryke C.R., Hsu N. (2015). Chromothriptic cure of WHIM syndrome. Cell.

[35] Li G.-M. (2008). Mechanisms and functions of DNA mismatch repair. Cell Res..

[36] Wallace S.S. (2014). Base excision repair: A critical player in many games. DNA Repair (Amst)..

[37] Misteli T., Soutoglou E. (2009). The emerging role of nuclear architecture in DNA repair and genome maintenance. Nat. Rev. Mol. Cell Biol..

[38] Polo S.E., Jackson S.P. (2011). Dynamics of DNA damage response proteins at DNA breaks: A focus on protein modifications. Genes Dev..

[39] Papadopoulos N., Lindblom A. (1997). Molecular basis of HNPCC: Mutations of MMR genes. Hum. Mutat..

[40] Lynch H.T., Snyder C., Casey M.J. (2013). Hereditary ovarian and breast cancer: What have we learned?. Ann. Oncol. Off. J. Eur. Soc. Med. Oncol..

[41] Economopoulou P., Dimitriadis G., Psyrri A. (2015). Beyond BRCA: New hereditary breast cancer susceptibility genes. Cancer Treat. Rev..

[42] Cortés-Ciriano I., Lee J.-K., Xi R., Jain D., Jung Y.L., Yang L., Gordenin D., Klimczak L.J., Zhang C.-Z., Pellman D.S. (2018). Comprehensive analysis of chromothripsis in 2,658 human cancers using whole-genome sequencing. bioRxiv.

[43] Ratnaparkhe M., Hlevnjak M., Kolb T., Jauch A., Maass K.K., Devens F., Rode A., Hovestadt V., Korshunov A., Pastorczak A. (2017). Genomic profiling of Acute lymphoblastic leukemia in ataxia telangiectasia patients reveals tight link between ATM mutations and chromothripsis. Leukemia.

[44] Keijzers G., Bakula D., Scheibye-Knudsen M. (2018). Monogenic Diseases of DNA Repair. N. Engl. J. Med..

[45] Mokrani-Benhelli H., Gaillard L., Biasutto P., Le Guen T., Touzot F., Vasquez N., Komatsu J., Conseiller E., Picard C., Gluckman E. (2013). Primary microcephaly, impaired DNA replication, and genomic instability caused by compound heterozygous ATR mutations. Hum. Mutat..

[46] Alderton G.K., Joenje H., Varon R., Borglum A.D., Jeggo P.A., O’Driscoll M. (2004). Seckel syndrome exhibits cellular features demonstrating defects in the ATR-signalling pathway. Hum. Mol. Genet..

[47] Chan T.A., Hermeking H., Lengauer C., Kinzler K.W., Vogelstein B. (1999). 14-3-3Sigma is required to prevent mitotic catastrophe after DNA damage. Nature.

[48] Canman C.E. (2001). Replication checkpoint: Preventing mitotic catastrophe. Curr. Biol..

[49] Roninson I.B., Broude E.V., Chang B.D. (2001). If not apoptosis, then what? Treatment-induced senescence and mitotic catastrophe in tumor cells. Drug Resist. Updat..

[50] Rosin M.P., German J. (1985). Evidence for chromosome instability in vivo in Bloom syndrome: Increased numbers of micronuclei in exfoliated cells. Hum. Genet..

[51] Bischof O., Kim S.H., Irving J., Beresten S., Ellis N.A., Campisi J. (2001). Regulation and localization of the Bloom syndrome protein in response to DNA damage. J. Cell Biol..

[52] Chan K.-L., North P.S., Hickson I.D. (2007). BLM is required for faithful chromosome segregation and its localization defines a class of ultrafine anaphase bridges. EMBO J..

[53] Honma M., Tadokoro S., Sakamoto H., Tanabe H., Sugimoto M., Furuichi Y., Satoh T., Sofuni T., Goto M., Hayashi M. (2002). Chromosomal instability in B-lymphoblasotoid cell lines from Werner and Bloom syndrome patients. Mutat. Res..

[54] Garcia A.M., Salomon R.N., Witsell A., Liepkalns J., Calder R.B., Lee M., Lundell M., Vijg J., McVey M. (2011). Loss of the bloom syndrome helicase increases DNA ligase 4-independent genome rearrangements and tumorigenesis in aging Drosophila. Genome Biol..

[55] Yamanishi A., Yusa K., Horie K., Tokunaga M., Kusano K., Kokubu C., Takeda J. (2013). Enhancement of microhomology-mediated genomic rearrangements by transient loss of mouse Bloom syndrome helicase. Genome Res..

[56] Killen M.W., Stults D.M., Adachi N., Hanakahi L., Pierce A.J. (2009). Loss of Bloom syndrome protein destabilizes human gene cluster architecture. Hum. Mol. Genet..

[57] Girard P.M., Foray N., Stumm M., Waugh A., Riballo E., Maser R.S., Phillips W.P., Petrini J., Arlett C.F., Jeggo P.A. (2000). Radiosensitivity in Nijmegen Breakage Syndrome cells is attributable to a repair defect and not cell cycle checkpoint defects. Cancer Res..

